# The Role of Free Radicals in the Aging Brain and Parkinson’s Disease: Convergence and Parallelism

**DOI:** 10.3390/ijms130810478

**Published:** 2012-08-21

**Authors:** Hemant Kumar, Hyung-Woo Lim, Sandeep Vasant More, Byung-Wook Kim, Sushruta Koppula, In Su Kim, Dong-Kug Choi

**Affiliations:** Department of Biotechnology, Konkuk University, Chungju 380-704, Korea; E-Mails: hemantbhave@gmail.com (H.K.); sksguddnek@hanmail.net (H.-W.L.); sandeepbcp@gmail.com (S.V.M.); kb62@lycos.co.kr (B.-W.K.); sushrutak@gmail.com (S.K.); kis5497@hanmail.net (I.S.K.)

**Keywords:** free radicals, aging, Parkinson’s disease, α-synuclein, mitochondrial dysfunction, nrf2

## Abstract

Free radical production and their targeted action on biomolecules have roles in aging and age-related disorders such as Parkinson’s disease (PD). There is an age-associated increase in oxidative damage to the brain, and aging is considered a risk factor for PD. Dopaminergic neurons show linear fallout of 5–10% per decade with aging; however, the rate and intensity of neuronal loss in patients with PD is more marked than that of aging. Here, we enumerate the common link between aging and PD at the cellular level with special reference to oxidative damage caused by free radicals. Oxidative damage includes mitochondrial dysfunction, dopamine auto-oxidation, α-synuclein aggregation, glial cell activation, alterations in calcium signaling, and excess free iron. Moreover, neurons encounter more oxidative stress as a counteracting mechanism with advancing age does not function properly. Alterations in transcriptional activity of various pathways, including nuclear factor erythroid 2-related factor 2, glycogen synthase kinase 3β, mitogen activated protein kinase, nuclear factor kappa B, and reduced activity of superoxide dismutase, catalase and glutathione with aging might be correlated with the increased incidence of PD.

## 1. Introduction

Chemical species with unpaired or an odd number of electrons are called free radicals. In biological systems, the term free radicals mostly refers to reactive oxygen species (ROS) and are oxygen centered [1,2]. Major ROS include superoxide anion (O_2_^−^), hydrogen peroxide (H_2_O_2_), and hydroxyl radical (·OH). Besides ROS, reactive nitrogen species (RNS), including nitric oxide (NO), peroxynitrite (NO_3_^−^), S-nitrosothiols also contribute to the generation of free radicals. Free radicals such as ROS and RNS arise as intermediates in many metabolic processes [3], are generated specifically as part of a cellular defense mechanism against invaded pathogens [4], and regulate several processes including glucose metabolism, cellular growth, and proliferation [5]. Superoxide radical and NO are the most commonly synthesized reactive species produced by NADPH oxidases and NO synthases, respectively [6]. These enzymes are highly active in the reproductive system, and ROS are involved in variety of functions, including elevation of intracellular Ca^2+^ concentrations, phosphorylation of specific proteins, activation of specific transcription factors, modulation of eicosanoid metabolism, stimulation of cell growth [7], and physiological mediators of control for several transcription factors [8]. Apart for beneficial effects, free radical causes lot of deleterious effects. ROS react with nucleic acids, proteins, and membrane lipids largely in a nonspecific manner, which may result in gene mutations, impairments or loss of enzyme activity, or altered cell membrane permeability, whereas RNS directly or indirectly lead to protein *S*-nitrosylation [9,10]. As a consequence of DNA being constantly attacked by free radicals, approximately 75,000–100,000 DNA damage events might occur in each cell per day [11,12]. Free radicals are deleterious in many ways, such as by damaging nucleobases or sugar units. ·OH is the most reactive species, and interacts with the C-8 position of guanine to form 8-hydroxyguanine, which is one of the most commonly found oxidized bases in DNA [13].

**Figure 1:**
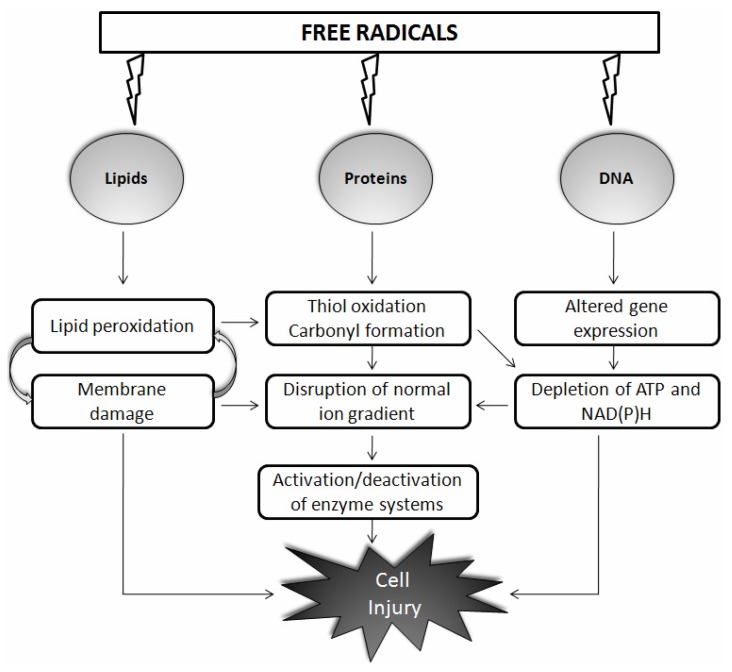
Schematic representation of the action of free radicals on biological molecules such as lipids, proteins, and DNA. Free radicals react largely in a nonspecific manner with nucleic acids, proteins, and membrane lipids and cause cell injury through various mechanisms as shown. Details are discussed in the main text.

The “free radical theory of aging”, published more than 50 years ago by Harman, states that the generation and accumulation of free radicals with aging results in oxidative damage to critical biological molecules such as DNA, proteins, and lipids [14] (Figure 1). Sixteen years later, Harman himself concluded that mitochondria are both the source and target of free radicals. This free radical theory of aging has become the mitochondrial free radical theory of aging, which is the most famous version of Harman’s theory [15]. Neural tissues have post-mitotic cells, and, moreover, their high oxygen consumption, lipid content, and metabolic activity make them more sensitive to oxidative damage than that of other tissues. It is difficult to quantify reactive species due to their highly evanescent and reactive nature; and evidence for disease comes from the detection of relatively stable products derived by the oxidation of cellular macromolecules. Increased immunoreactivity to indices of oxidative stress occurs in humans with physiological aging [16], and pathological aging further exacerbates this effect [17]. During aging or under pathological states, the oxidation frequency of biological targets increases as repair processes slow down and detection of oxidized proteins, lipids, and DNA becomes more apparent. Notably, up to 50% of proteins may be oxidized in an 80-year-old human [18]. It was initially thought that aging could be manipulated with the use of antioxidants, and this seems to be attractive approach. Some studies have reported large changes in longevity after overexpression of antioxidants [19], whereas others failed to see any change [20]. In invertebrates, the use of antioxidants to increase longevity has been contradictory [21]. In a similar approach, supplementation, induction, or overexpression of antioxidants has continuously failed to significantly increase maximum life span in mammals [22].

Parkinson’s disease (PD), the most frequent neurodegenerative condition after Alzheimer’s disease (AD), is characterized by degeneration of dopaminergic neurons in the substantia nigra pars compacta (SNpc) and loss of striatal dopamine content [23,24]. Although several factors have been proposed for the pathogenesis of PD, oxidative stress via the generation of free radicals is one of the major contributors. In the pathology of neurodegenerative disorders, the generation of free radicals, particularly ROS and RNS, are harmful, as they affect proteins, lipids, and nucleic acids [9,10]. Initial evidence for the existence of oxidative stress in PD came from reports based on post-mortem analyses of brain tissue from patients with PD that demonstrated increased levels of oxidized proteins, lipids, and nucleic acids [25,26].

**Figure 2:**
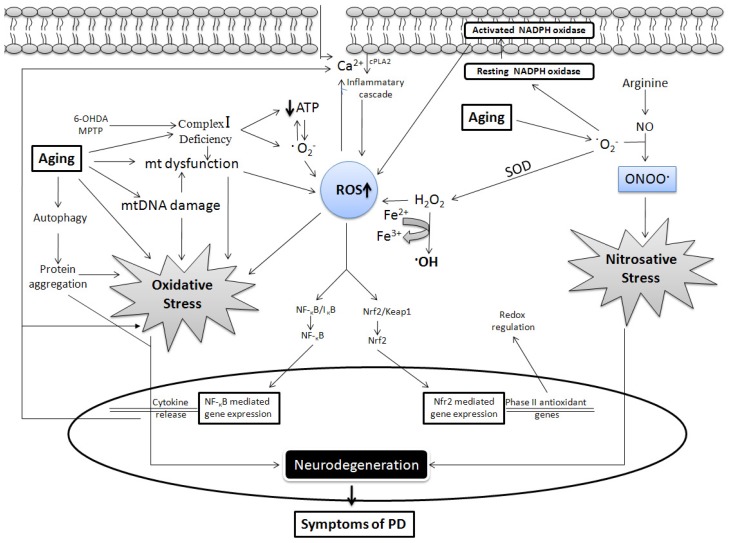
Generation of free radicals in aging and Parkinson’s disease (PD). A major source of free radicals is mitochondria (mt), mitochondrial complex inhibition either by toxins or aging hampers the mitochondrial respiratory chain, which causes incomplete oxygen reduction, thereby generate reactive species including deleterious superoxide anion (O_2_^−^) which is converted to hydrogen peroxide (H_2_O_2_) and then finally to hydroxyl radical (·OH) through the Fenton reaction which involves Fe^2+^ or Cu^2+^ (not shown). ·OH is a potent inducer of membrane lipid peroxidation. Oxyradicals can also be generated in response to calcium influx. Nitric oxide (NO) interacts with O_2_^−^ to form peroxynitrite (NO_3_^−^)_,_ Reactive oxygen species (ROS) and reactive nitrogen species (RNS) contribute to oxidative and nitrosative stress, respectively, which finally causes neurodegeneration. Mitochondrial activity is lost or mitochondrial DNA is damaged during aging, which could lead to generation of ROS. Cytoprotective pathways are activated with the generation of free radicals. Activation of the Nrf2 pathway provides protection by regulating redox balance, whereas the NF-κB pathway causes increased cytokine release which is included in positive feedback to initiate the inflammatory cascade and also through influx of calcium ions inside the extracellular space.

It is now well established that free radicals play a crucial role in aging [27,28], and aging is considered as one of strongest risk factors for PD [29,30]. The prevalence of PD increases with age, and occurs in approximately 1% of people >60 years, which increases to about 4% in individuals >85 years [31,32]. There is no full-proof theory that links age with these two, although substantial evidence can be used to extrapolate the relationship. Moreover, a 5–10% linear fallout of dopamine neurons occurs per decade of aging [33], and PD was once proposed to be a form of accelerated aging [34]. Due to advances in research and development in the health sector, average life span is increasing. and it has been argued that the incidence of PD will rise in the coming years [35]. In contrast, the rate and intensity of neuronal loss in patients with PD is more marked that that of physiological aging, and clinical signs of PD are detected when 50% of nigral neurons and 80% of striatal dopamine are lost [36]. In this review we enumerate the common link between aging and PD at the cellular level with special reference to oxidative damage caused by free radicals, which includes mitochondrial dysfunction, dopamine auto-oxidation, α-synuclein aggregation, glial cell activation, alterations in calcium signaling, and excess free iron. Moreover, neurons encounter more oxidative stress with advancing age, as defense mechanisms do not function properly. An alteration in transcriptional activity of various pathways such as nuclear factor erythroid 2-related factor 2 (Nrf2), glycogen synthase kinase 3β (GSK-3β), nuclear factor kappa B (NF-κB), and reduced activity of superoxide dismutase (SOD), catalase and glutathione (GSH) occurs with aging, which may be correlated with the increased incidence of PD (Figure 2).

## 2. Common Link between PD and Aging: Role of Free Radicals

### 2.1. Mitochondrial Dysfunction

Mitochondrial oxidative phosphorylation (OXPHOS) is the primary source of high-energy compounds in the cell. Mitochondrial dysfunction leads to reduced ATP production, increased oxidative stress, altered mitochondrial morphology, impaired calcium buffering, damage to mitochondrial DNA (mtDNA), and alterations in mitochondrial fission and fusion, eventually leading to cell death [37]. Mitochondria are both targets as well as important sources of free radicals. A major theory of aging states that aging is a direct consequence of accumulated damaged mtDNA produced by ROS and other related free radicals generated during the course of OXPHOS [15]. Mitochondrial complex I alterations are a major source of ROS generation in patients with PD. An inhibited mitochondrial complex hampers the mitochondrial respiratory chain, which causes incomplete oxygen reduction, thereby generating reactive species including deleterious O_2_^−^ [38] that is further converted to NO_3_^−^ and, finally, to ·OH through the Fenton reaction [39]. Thus, dysfunctional mitochondria are the primary intracellular source of ROS contributing to oxidative stress-mediated neurodegeneration in PD models [23,40]. It has long been known that somatic mtDNA mutations are responsible for some aspects of the aging process [41]. Interestingly, a very high absolute prevalence of mtDNA deletions in neurons from aged SNpc, and significant differences in mtDNA deletions between old and young tissues have been reported [42]. In another study, SN neurons from aged controls and individuals with PD was compared, and high levels of deleted mtDNA were detected, suggesting that somatic mtDNA deletions are important in the selective neuronal loss observed in brain aging and in PD [43]. Collectively, SN dopaminergic neurons in both aged individuals and patients with PD harbor high levels of mtDNA deletions (up to 60% mtDNA deletion load), which are associated with cytochrome *c* oxidase (complex IV) dysfunction [42,43]. Disruptions in OXPHOS are believed to act as primary or secondary contributors to neuronal loss in PD, and OXPHOS complexes contribute to the overall decline in mitochondrial bioenergetics. mtDNA is particularly important, as it encodes for subunits that contribute to all OXPHOS complexes except complex II [44]. Interestingly, genetic ablation of p66Shc, a pro-apoptotic mitochondrial ROS-producing protein, extends the lifespan of mutant mice [45]. Another study showed that overexpression of the enzyme peptide methionine sulfoxide reductase A (MSRA), which is produced predominantly in the nervous system, markedly extends lifespan of the fruit fly *Drosophila*. Furthermore, MSRA transgenic animals are more resistant to paraquat-induced oxidative stress, and the onset of senescence-induced decline in general activity level and reproductive capacity is markedly delayed. These results suggest that oxidative damage is an important determinant of lifespan, and that MSRA might play an important role in increasing the lifespan of other organisms including humans [46].

An enzyme called mtDNA polymerase Polg A, involved in copying and proofreading mtDNA, eliminates errors made during replication and participates in DNA repair processes. Interestingly, mutant mice carry mutations in Polg A (hampering proofreading activity), show extensive mtDNA mutations in almost all tissues, decreased activity of enzymes involved in the respiratory chain and in the production of ATP, increased apoptosis, accelerated aging, and reduced lifespan [47,48]. These findings suggest that mtDNA mutations acquired during normal aging could accelerate the aging process through increased ROS generation. Furthermore, Polg A mutations are associated with parkinsonism in humans [49]. In one study, age-dependent regulation of gene expression in the human brain was investigated in 30 individuals ranging from 26 to 106 years in age. Interestingly, a downregulation of genes involved in synaptic plasticity, vesicular transport, and mitochondrial function occurred in individuals >76 years, which was accompanied by induction of stress response, antioxidant, and DNA repair genes as well as marked DNA damage. Using small interfering RNA, authors mimicked the reduced expression of mitochondrial genes and found increased DNA damage in vulnerable nuclear genes that correlated with dysfunctional mitochondria as a ROS source in the aging brain [50]. Thus, it would be reasonable to anticipate that age-dependent mitochondrial dysfunction and ROS generation impairs global OXPHOS and general energy metabolism, either inferring a risk for PD or exacerbating its symptoms.

Apart from environmental factors and aging, redox sensitive genes are also known to increase the sensitivity of cells to oxidative stress. Mutations in *PARK7*, which encodes DJ-1, are oxidatively damaged and increase significantly in brains of patients with sporadic PD [51–53], or sometimes represent a rare cause of early-onset familial PD [54]. Exposure to oxidative toxins or over-oxidation of DJ-1 with age might lead to inactivation of DJ-1 function, suggesting a role in susceptibility to sporadic PD [55]. Interestingly, aged DJ-1 knockout (KO) mice do not show significant differences in protein nitration, nucleic acid oxidation, or lipid peroxidation in the SN [56]. Mutations in parkin [54] and PINK1 [54,57] account for early-onset familial PD. Disruption of parkin contributes to the etiology of PD either by disrupting normal function of the ubiquitin proteasome system in the clearance of aggregated proteins or by disabling a mitochondrial protective mechanism mediated by a signaling function of parkin, contributing to mitochondrial dysfunction [54]. As with parkin, loss-of-function of PINK1 leads to decreased mitochondrial protection against oxidative stress, causing enhanced mitochondrial dysfunction [58]. Overexpression of parkin and PINK1 rescues the α-synuclein*-*induced PD-like phenotype in *Drosophila melanogaster*, presumably through targeting the α-synuclein protein for degradation [59,60]. Moreover, no significant differences in protein oxidation have been found in different brain structures of parkin KO mice at the ages of 2, 3, 12, and 22 months [61–63]. However, when the entire brain was analyzed, increased protein oxidation occurred in parkin KO mice at 18–20 months [61] and lipid peroxidation occurred in aged parkin KO mice [61]. Mutations in the parkin gene [64,65] are the most common genetic risk factors for early-onset PD [66–68] with age at onset ≤ 45 or 55 years. Cases with parkin mutations with age at onset > 70 years have also been reported [68–70]. But, the frequency of parkin mutations may be as high as 49%, in cases of age at onset ≤45 years in families with an autosomal recessive mode of inheritance, whereas the reported range is 15–18% in cases without a family history of PD [67,71]. An inverse correlation has been observed in age at onset and frequency of parkin mutations in both the familial [66] and sporadic [67] forms of PD. Loss of function of DJ-1, parkin, and PINK1 leads to decreased mitochondrial protection against oxidative stress, causing enhanced mitochondrial dysfunction [58,72].

### 2.2. Role of Dopamine

Dopamine neurons are exposed to ROS and RNS throughout their lifespan from the metabolism of cytosolic dopamine itself. Dopamine, as a relatively unstable molecule in nature, undergoes auto-oxidation metabolism in the nigrostriatal tract system thereby producing ROS [73], and auto-oxidation itself may increase with age [74]. The unstored cytosolic fraction undergoes spontaneous or monoamine oxidase B (MAO-B)-mediated degradation to form 3, 4-dihydroxyphenylacetic acid (DOPAC) and homovanillic acid (HVA) as major metabolites, as well as O_2_^−^, H_2_O_2_, and dopamine quinones as minor metabolites [39,75]. MAO-B activity increases with age [76], which results in decreased availability of catecholamines in the synaptic cleft [77]. Oxidative deamination of primary MAO produces NH_3_ and H_2_O_2_ with established or potential toxicity [78]. Bradykinesia (declining motor functions), a characteristic hallmark of PD, is also seen robustly during physiological aging. This characteristic feature is a reflection of qualitative and quantitative changes in dopamine function in the SN and striatum [79] and is correlated with the decline in dopamine levels during aging and in PD. Initial evidence showed that dopamine levels decline by 50–60% during advanced normal aging [80,81], whereas loss of dopamine neurons in patients with PD is 80–90% in the SN and 40–50% in the ventral tegmental area (VTA) [82]. It has also been suggested that dopamine deficiency sufficient to provoke PD symptoms is expected in normal aging by 110–115 years [81]. Moreover, the mesolimbic system is affected more during aging, whereas the nigrostriatal system is the main target in PD [83]. Dopamine metabolites (e.g., DOPAC, HVA) decline with age in rat nigrostriatal, mesocortical, and hippocampal regions [84]. The main cellular defense against unstored dopamine is the recapture of dopamine released into the synapse by the dopamine transporter (DAT), followed by sequestration into synaptic vesicles by vesicular monoamine transporter 2. However, a significant decline in DAT occurs with age compared to that in healthy normal volunteers [85], and cytosolic dopamine produces H_2_O_2_ and ·OH causing damage to cells [86,87].

In one study, young (6 months) and middle-aged (15 months) rats were chronically treated with dopamine in the SN and examined for changes in motor function and histology. It was observed that dopamine-induced toxicity was age dependent, and that middle-aged animals showed more impairment following a dopamine injection than that of younger animals [88]. An age-related loss in nigrostriatal axons was observed, and a steady decrease in dopamine uptake sites was seen. In contrast, D1 and D2 dopamine receptor concentrations and their high-agonist affinity sites are not affected by the aging process [89]. Another study examined neurochemical, morphological, and functional markers of the nigrostriatal dopamine system in young, intermediate-aged, and old squirrel monkeys. As a result, a significant age-related loss of dopamine was observed in the SN (70%) and the putamen (30%) but not in the caudate. However, nigrostriatal dopamine loss did not appear to be a consequence of age-related loss of dopaminergic nigral neurons, as the number of tyrosine immunoreactive cells was not significantly different among the three age groups. Additionally, the region-selective loss of dopamine correlates with PD, thereby raising the possibility of a relationship between physiological aging and development of the disease [90]. Additional evidence for the involvement of free radicals in dopamine metabolism is tyrosine hydroxylase (TH), the rate-limiting enzyme for dopamine synthesis. Interestingly, TH is inhibited by oxidation [91]. Around 55% loss in TH activity in the SN of rats is observed by 24 months of age, which is associated with a 59% increase in protein carbonyls. Furthermore, H_2_O_2_-decreased TH activity was in direct proportion to protein carbonylation [92].

### 2.3. α-Synuclein

Proteinacious fibril accumulation is a common neuropathological feature of neurodegenerative diseases. α-Synuclein is a hallmark of PD and is modified by nitration of tyrosine residues. In the presence of reactive oxygen intermediates, NO forms RNSs capable of modifying tyrosine residues in proteins to form 3-nitrotyrosine [93]. In native soluble form, α-synuclein may modulate synaptic plasticity [94] and dopaminergic neurotransmission [95,96]. Aggregation of α-synuclein into protofibrils causes loss in its normal function and impairs dopaminergic neutrotransmission. This aggregation is induced by the Cu-Zn-SOD/H_2_O_2_ system via the generation of ·OH [97], and/or ROS produced by dopamine metabolism [98,99]. α-Synuclein is found in cerebrospinal fluid (CSF) from patients with PD and also in age-related controls [100]. The levels of α-synuclein protein in human SN increase with aging, and aging is associated with increased levels of oxidatively modified α-synuclein but is rarely present in inclusions typical of PD [101–103]. A recent study assessed nigral neuronal loss and α-synuclein immunopositive Lewy bodies in >2500 individuals and brains from 744 deceased participants without PD. They concluded that nigral pathology is common in individuals without PD and may contribute to loss of motor function in old age [104]. A comparative study between young and old normal humans and rhesus monkeys revealed robust age-related increases in the α-synuclein protein within individual nigral areas but not in the VTA in either species. The age-related increase in nigral α-synuclein was non-aggregated and strongly correlated with age-related decreases in TH. β-synuclein, a non-pathogenic analogue of α-synuclein, does not show age-related changes. Moreover, distinct staining for α-synuclein is apparent in young, middle-aged, and aged subjects [101]. Thus, accumulation of α-synuclein within aging nigral neurons is strongly correlated with a loss of dopamine phenotype. Interestingly, decreased DAT activity in the aged brain is related with lower α-synuclein levels in the plasma membrane, probably by NO induced transporter alterations [105]. One study examined 157 brains from a geriatric hospital, and anti-phosphorylation sites were observed in 40 cases. Immunohistochemistry revealed four novel types of pathology. These findings suggest that Lewy body (LB)-related pathology initially involves the neuronal perikarya, dendrites, and axons, causes impairment in axonal transport and synaptic transmission, and later leads to the formation of LBs, a hallmark of functional disturbance long before neuronal cell death [106].

Interestingly, α-synuclein overexpression increases intracellular ROS levels and susceptibility to dopamine [107]. Oxidative stress can also induce α-synuclein aggregation [108]. An *in vivo* study demonstrated that the ultimate origin of free radical damage in patients with PD could be the production of H_2_O_2_ by α-synuclein. α-Synuclein and iron accumulation in the SN could establish the favorable local conditions required for α-synuclein-mediated H_2_O_2_ formation and its conversion to ·OH via the Fenton reaction; thus, leading to death of vulnerable nigral neurons [109]. Chaperones are ubiquitous, highly conserved proteins [110] that either assist in folding of newly synthesized proteins or sequester damaged proteins for future refolding. α-Synuclein also becomes phosphorylated, leading to misfolding and aggregation in patients with PD [111]. Dysfunction in the ubiquitin proteasome system leads to protein accumulation [112,113]. The ubiquitylation of α-synuclein within LBs suggests a connection with proteasomes, and α-synuclein is a known substrate of chaperone-mediated autophagy involving lysosomes [114–116]. Thus, synucleinopathy may be a product of impaired processing of abnormal forms of α-synuclein and/or abnormal levels of α-synuclein by the intracellular proteasome and lysosome systems.

### 2.4. Glial Cells

#### 2.4.1. Microglia

Microglia are the primary resident immune cells of the central nervous system (CNS), and play a critical role in neurodegenerative diseases [117], as well as in age-related macular degeneration [117,118]. Activated microglia are the most abundant source of free radicals in the brain by releasing free radicals such as O_2_^−^ and NO [119]. Moreover, over-activated microglia contribute to neurodegenerative processes by producing various neurotoxic factors including free radicals and proinflammatory cytokines [120], or via free radicals generated by activated NAD(P)H oxidases [121]. CD11b, a beta-integrin marker of microglia, increases during microglial activation, upregulation of CD11b in microglia is redox sensitive, and ROS up-regulates CD11b via NO [122]. Initially, one study suggested that degeneration of dopaminergic neurons in PD is associated with massive microglial activity in the SNpc [123], but later presence of activated microglia in the putamen, hippocampus, transentorhinal cortex, cingulate cortex, and temporal cortex has been reported [124]. A recent *in vivo* study indicated that microglial activation in patients with PD is anatomically widespread in the pons, basal ganglia, and frontal and temporal cortical regions, and that the level of microglial activation is independent of clinical severity [125]. Microglia maintain microenvironmental homeostasis during brain injury by migrating to the lesion site, clearing cellular debris, and producing pro-inflammatory cytokines such as tumor necrosis factor alpha (TNFα), interleukins 1 beta (IL-1β) and 6 (IL-6), and anti-inflammatory cytokines such as interleukin 10 (IL-10) and transforming growth factor beta 1 (TGFβ1) [126]. Converging evidence shows that aging microglia also have lipofuscin granules, decreased process complexity, altered granularity, and increased mRNA expression of both proinflammatory (TNFα, IL-1β, and IL-6), and anti-inflammatory (IL-10 and TGFβ1) cytokines [127]. Microglia can be neuroprotective or neurotoxic to dopaminergic neurons depending on age [128]. Moreover, pro-inflammatory cytokines chronically increase in the aging brain [129], and aged microglia in the resting state have significantly smaller and less branched dendritic arbors as well as slower process motility, which compromises their response to injury [130]. 1-methyl-4-phenyl-1,2,3, 6-tetrahydropyridine (MPTP) administration in elderly mice results in severe and persistent microglial activation [131]. Activated microglia release O_2_^−^ [132,133], which acts as a reducing agent and can cause the release of iron from ferritin, which further provokes lipid peroxidation [134]. Dystrophic microglia express high levels of ferretin, an iron storage protein [135], suggesting their role during attack by free radicals.

#### 2.4.2. Astrocytes

Astrocytes are the most abundant cell type in the brain; they are electrically inert and derived from the same progenitors as neurons. Astrocytes play a significant role in brain injury, as they respond to injury in conjunction with microglia through a process called reactive gliosis or astrogliosis [136]. Glial fibrillary acidic protein (GFAP) is the most commonly used marker of mature astrocytes in the CNS and has been the most common change observed during aging [137]. Vimentin is another intermediate filament in astrocytes, and its expression also increases with aging [138]. Approximately, 20% increase in astrocytes occurs in the aged cortex and other brain regions [139–141]. As discussed previously, oxidative stress and inflammatory responses are associated with aging and PD, and the increase in GFAP in aged astrocytes may be the result of these responses. There are several physiological ways in the body to deal with ROS, as the brain is equipped with defense systems such as the antioxidant enzymes SOD, glutathione peroxidase, and catalase as well as the antioxidant GSH [142]. The concentration of GSH in astrocytes (~3.8 mM) is thought to be higher compared to that in neurons (~2.5 mM) [143], probably as a result of higher specific activity of the γ-glutamylcysteine synthetase (a rate-limiting enzyme in GSH biosynthesis) in astrocytes [144]. Thus, astrocytes are thought to be the major contributor of GSH and help in removal of ROS. The GSH content in the SN of patients with PD is significantly reduced (~40%), making them more vulnerable to the deleterious effect of free radicals [145].

O’Callaghan and Miller [146] reported an elevation in GFAP expression with age throughout the brain, with the largest increase observed in corpus striatum. TH-positive neuron number is unaltered in the SN with increasing age, whereas the ratio of TH-positive neurons to GFAP-positive glial cells decreases slightly with increasing age [147]. Interestingly, selective expression of a mutant α-synuclein protein in astrocytes *in vivo* results in SN neurodegeneration, behavioral dysfunction, and shortened lifespan [148]. When mutant α-synuclein protein is expressed under control of the human α-synuclein promoter, which is expressed in fewer astrocytes and more in neurons, it results in 100% death of animals after 3 months [149,150], suggesting that astrocytic dysfunction might be crucial for initiating disease and not just for downstream neurodegenerative effects.

A recent study demonstrated that H_2_O_2_ rapidly induces the phosphorylation, nuclear translocation, and binding of signal transducer and activator of transcription 6 (STAT6) to the promoter of the cyclooxygenase-2 gene, resulting in the expression and subsequent release of prostaglandin PGE_2_ and PGI_2_ in primary rat brain astrocytes. Furthermore, STAT6 in astrocytes is much more sensitive to ROS*-*dependent phosphorylation than that of microglial STAT6, and astrocytes sense H_2_O_2_ by rapidly phosphorylating the STAT6 transcription factor, a response not observed in microglia. Therefore, astrocytes, through this STAT6-activation mechanism, could function as ROS sensors in the brain, and this phosphorylation is induced by generators of other ROS and RNS [151].

### 2.5. Regulation of Calcium

Calcium (Ca^2+^) signaling can be altered by oxidants and this alteration can modify essential pathways. After the mitochondrial free radical theory of aging was introduced, the Ca^2+^ hypothesis of brain aging was formulated in the 1980s, which was based on a limited number of observations in processes that are regulated by Ca^2+^ alterations [152]. In the 1990s, major advances in identifying biological markers of Ca^2+^ dependent processes were made that could change during aging. In most neurons, opening of Ca^2+^ channels is a uncommon event, and it occurs during a very brief action potential [153]. SNpc dopaminergic neurons have an unusual physiological phenotype, as they exhibit pacemaker activity even in the absence of excitatory input. Most neurons utilize Na^+^ channels to maintain action potentials, whereas SN neurons rely upon l-type Ca^2+^ channels to govern pacemaking [154,155]. The magnitude of Ca^2+^ influx and sustained demand of OXPHOS appear to be much larger in SNpc neurons that that of other neurons, making them more vulnerable to oxidative damage and aging. Interestingly, dopamine neurons in the VTA and the olfactory bulb, both of which employ Na^+^ channels to generate pacemaking [156,157], are spared in human patients and animal models of PD [158,159], suggesting a link between selective vulnerability due to Ca^2+^ pacemaking instead of dopaminergic transmission. Mitochondria and the endoplasmic reticulum are the principal organelles involved in sequestering Ca^2+^ in neurons [160,161], and dysfunction in these organelles that occurs during aging and PD could result in the generation of oxidative stress. It is generally accepted that oxidants cause a rapid increase in Ca^2+^ concentrations in the cytoplasm of diverse cell types [162,163]. Oxidants such as H_2_O_2_ cause a sustained elevation in cytosolic Ca^2+^, which is not observed in Ca^2+^-free medium, suggesting that severe oxidative stress causes Ca^2+^ uptake by cells from the extracellular space [162]. An increase after hyperpolarization during aging is partially related to Ca^2+^ influx through l-type Ca^2+^ channels as well as changes in Ca^2+^ buffering [164].

### 2.6. Iron

Iron plays a key role in supporting systems responsible for myelination and the synthesis of several neurotransmitters in the CNS. Excess free iron generates oxidative stress because of its interaction with H_2_O_2_, and iron localization correlates with ROS in areas that are prone to neurodegeneration [165]. The toxicity of O_2_^−^ and H_2_O_2_ arises from their iron-dependent conversion into the extremely reactive ·OH (Haber–Weiss reaction), which causes severe damage to membranes, proteins, and DNA [166]. The rank order of total iron distribution in normal brain tissue is globus pallidus > putamen > SN > caudate nucleus > cerebral cortex = cerebellum [167]. Iron is most abundant in areas that are rich in dopaminergic neurons, namely, the globus pallidus, putamen, and SN of the basal ganglia [167]. Iron is stored primarily in glial cells where it is bound to storage protein ferritin [168]. There is an age-dependent increase in iron storage in the brain and an increase in the proportion of iron stored in ferritin [169,170]. Iron accumulation occurs in PD, as iron content of the SN is elevated compared to that in aged-matched controls with an increase in the Fe (III)/Fe (II) ratio from 2:1 to 1:2 [171]. Aging is associated with disturbances in iron metabolism and regulation in rodents [50,172], as well as in humans [131,173]. Free radicals are constantly generated in the brain as by-products of the oxidation/reduction reactions required by cellular metabolic processes. These reactions are catalyzed by transition metals such as iron and are more likely to occur in brain regions with a high concentration of these elements. Hence, any regional increase in brain iron concentration may increase the potential for local free-radical formation and lipid peroxidation [174].

As pointed out earlier, the region-specific increase in iron content could increase the probability of free radical formation in areas rich in iron. Physiological aging is associated with a significant increase in iron content in both the putamen and the caudate and, thereby, an increased risk for development of neurodegenerative disorders associated with aging. The clinical importance of this increase in striatal iron content in association with aging may be directly related to its involvement in free-radical generation. In the brain, ·OH reacts with membrane lipids, starting the chain reaction of lipid peroxidation, which, in turn, results in extensive membrane damage, ultimately leading to cell degeneration [175]. A comparative analysis of iron-related neuronal vulnerability performed in two brainstem nuclei, the locus coeruleus (LC) and SN, known targets in PD and age-related disorders, revealed that LC neurons are comparatively less affected with a variable degree of involvement than that of SN neurons. Moreover, iron content in the LC is much lower than that in the SN, and the ratio of heavy-chain ferritin iron in the LC is higher than that in the SN. These findings suggest that the iron mobilization and toxicity is lower in LC neurons than that in the SN and that it is efficiently buffered by neuromelanin. Thus, the more extensive damage occurring in the SN could be related to higher iron content [176]. The identification of iron regulatory proteins 1 and 2 (IRP1 and IRP2) in various regions of rat and mice brain including the striatum and SN strengthens the role of iron in neurodegenerative diseases including PD [177]. Mitochondrial complex I inhibitors such as MPTP and 6-OHDA, result in oxidative stress as a consequence of deregulation of mitochondrial iron and glutathione [178]. Iron chelators such as desferal [179] and VK-28 [180] show neuroprotective effects in neurotoxin models of PD.

### 2.7. Defective Mechanism

To counteract the stress caused by these free radicals, cells have developed adaptive, dynamic programs to maintain cellular homeostasis through a series of antioxidant molecules and detoxifying enzymes that can provide control by quick removal or detoxification. Nrf2 is one such major pathway that responds to reactive species by activating phase II detoxification enzymes at the transcriptional level [181,182]. When the redox balance is more toward the oxidative side, as occurs during attack by electrophilic and/or oxidative stimulus, Nrf2 is released from Kelch ECH-associated protein 1 (Keap1), is translocated to the nucleus, and binds with antioxidant response elements (ARE) in the promoter region of its target genes, thereby inducing a battery of cytoprotective genes and antioxidative enzymes [181,182]. The Nrf2 pathway is involved in the pathogenesis of PD [183,184], and its activity declines with age [185]. Mechanisms that decrease Nrf2 transcriptional activity interfere with removal/detoxification of free radicals. GSK-3β, a serine/threonine kinase, mediates Nrf2 phosphorylation and prevents nuclear localization, inhibits the transcriptional activity, and blocks the antioxidant and cytoprotective functions of Nrf2 [186]. Long-term activation of GSK-3β under conditions of persistent oxidative stress results in downregulation of Nrf2. Increased GSK-3β activity have been reported with aging [187] and in *in vitro* and *in vivo* models of PD [188,189]. The mitogen-activated protein kinase (MAPK) members, such as extracellular signal-related kinases (ERK), c-Jun *N*-terminal kinases (JNK), and p38, are involved in regulation of the ARE [190]. MAPK is also activated in PD models and is implicated in the mechanism of neuronal cell death [191]. Both JNK and ERK contribute directly to mitochondrial dysfunction by suppressing oxidative respiration in various experimental models of PD [192]. Age-related decreases in resistance to oxidative stress might result from lower expression of antioxidant enzymes, which are regulated by Nrf2 transcriptional activity and its mediators, MAPKs [193]. Moreover, elevated and sustained expression of stress-response signaling pathways (p38 MAPK, SAPK/JNK) is a major physiological characteristic of aged tissues [194]. p38 MAPK activation leads to mitochondrial ROS generation and sustained elevation of p38 MAPK activity in aged tissue promotes aging characteristics [195].

Several other pathways such as nuclear factor (NF-κB) are involved in PD and aging. The expression of many of NF-κB genes increases with age [196,197] and plays an important role in PD [198–200]. Promoter regions of proinflammatory molecules contain the DNA binding site for NF-κB and inhibiting NF-κB activation reduces the induction of proinflammatory molecules [201]. NF-κB drives the transcription of several proinflammatory molecules including inducible nitric oxide synthase, TNFα, and IL-1β in microglia and astroglia. These molecules play an important role in the loss of dopaminergic neurons in MPTP-intoxicated mice and patients with PD [198–200]. Moreover, the NF-κB components p52 and p65 but not p50 are clearly higher in nuclear extracts of old rodents compared to those of young ones [196,197]. SOD catalyzes the dismutation of O_2_^−^ into O_2_ and H_2_O_2_ and provides an important antioxidant defense mechanism. An increase in the level of Mn-dependent SOD occurs in the SN and CSF of patients with PD [202]. Moreover, total SOD and Mn SOD activities also increase with age in rats [203]. Another important antioxidant defense mechanism is GSH, also referred to as the master antioxidant, which can neutralize any type of chemical and scavenge free radicals. GSH is the earliest known indicator of nigral depletion, and the magnitude of GSH depletion is correlated with disease severity [204]. Moreover, a significant increase in oxidized glutathione turnover occurs in PD [205]. Glutamate cysteine ligase (GCL) catalyzes the first and rate-limiting step of *de novo* GSH synthesis, making it a major determinant of overall GSH synthetic capacity [206–208]. Reduced levels of GCL are observed throughout the brain as a consequence of aging [209]. In addition, mutant mice overexpressing the antioxidant enzyme catalase, specifically in the mitochondria, exhibit reduced accumulation of mtDNA mutations [210], increased lifespan [211], and more resistance to MPTP-induced dopaminergic cell death [212], suggesting a role for catalase in counteracting oxidative stress.

## 3. Conclusions

A progressive accumulation of damaged biomolecules and impaired energy metabolism occurs during aging and PD that promotes dysfunction of various metabolic processes and signaling pathways. As reviewed here, aging and PD share common features that are interlinked with the generation of free radicals; free radicals form a feedback loop such that separating the two processes is difficult. Neural tissue encounters a cumulative burden of oxidative and metabolic stress; moreover, neural tissues have post-mitotic cells, high oxygen consumption; lipid content and metabolic activity making them more vulnerable to the deleterious effects of free radicals. Convergence and parallelism occurs between aging and PD. For example, post-mortem analyses of brain tissue from PD patients were found to have increased levels of oxidized proteins, lipids and nucleic acid. Notably, up to 50% of proteins may be oxidized in an 80-year old human. Furthermore, Initial evidence have showed that dopamine levels decline by 50–60% during advanced normal aging; whereas clinical signs of PD are detected when 50% of nigral neurons and 80% of striatal dopamine are lost. Moreover, bradykinesia (declining motor function), a characteristic hallmark of PD, is also seen quite often during physiological aging. This characteristic feature is a reflection of qualitative and quantitative changes in dopamine function in the SN and striatum and is correlated with declining dopamine levels during both aging and PD. When compared on anatomical site, mesolimbic system is affected more during aging, whereas the nigrostriatal system is the main target in PD. Deficiency of dopamine sufficient to provoke PD symptoms would be expected in normal aging of 110–115 years. Though, several common factors can be seen in PD and aging, still most of the therapeutic approaches (Ca^2+^ channel blockers, dopamine agonists, iron chelators, and antioxidants) ameliorate PD symptoms but do not reverse the aging process. Several factors contribute to the generation of free radicals either directly (mitochondrial dysfunction and dopamine auto-oxidation) or indirectly (α-synuclein, glial activation, free iron, and altered calcium signaling). Physiological mechanisms to counteract oxidative stress are diminished during aging; thus, making individuals more prone to neurodegenerative diseases such as PD. It is still debatable that age-related changes in the brain reflect aging-associated neurodegenerative diseases rather than the aging process itself.

## References

[1] Brand M.D., Affourtit C., Esteves T.C., Green K., Lambert A.J., Miwa S., Pakay J.L., Parker N. (2004). Mitochondrial superoxide: Production, biological effects, and activation of uncoupling proteins. Free Radic. Biol. Med.

[2] Turrens J.F. (2003). Mitochondrial formation of reactive oxygen species. J. Physiol.

[3] Thannickal V.J., Fanburg B.L. (2000). Reactive oxygen species in cell signaling. Am. J. Physiol. Lung. Cell. Mol. Physiol.

[4] Forman H.J., Torres M. (2002). Reactive oxygen species and cell signaling: Respiratory burst in macrophage signaling. Am. J. Respir. Crit. Care Med.

[5] Rhee S.G. (1999). Redox signaling: Hydrogen peroxide as intracellular messenger. Exp. Mol. Med.

[6] Bedard K., Krause K.H. (2007). The NOX family of ROS-generating NADPH oxidases: Physiology and pathophysiology. Physiol. Rev.

[7] Kaul N., Forman H.J., Rhodes C.R. (2000). Reactive Oxygen Species in Physiology and Toxicology: From Lipid Peroxidation to Transcriptional Activation.

[8] Nordberg J., Arner E.S. (2001). Reactive oxygen species, antioxidants, and the mammalian thioredoxin system. Free Radic. Biol. Med.

[9] Valko M., Rhodes C.J., Moncol J., Izakovic M., Mazur M. (2006). Free radicals, metals and antioxidants in oxidative stress-induced cancer. Chem. Biol. Interact.

[10] Finkel T. (2003). Oxidant signals and oxidative stress. Curr. Opin. Cell Biol.

[11] Lindahl T., Nyberg B. (1972). Rate of depurination of native deoxyribonucleic acid. Biochemistry.

[12] Lindahl T., Karlstrom O. (1973). Heat-induced depyrimidination of deoxyribonucleic acid in neutral solution. Biochemistry.

[13] Devasagayam T.P., Steenken S., Obendorf M.S., Schulz W.A., Sies H. (1991). Formation of 8-hydroxy(deoxy)guanosine and generation of strand breaks at guanine residues in DNA by singlet oxygen. Biochemistry.

[14] Harman D. (1956). Aging: A theory based on free radical and radiation chemistry. J. Gerontol.

[15] Harman D. (1972). The biologic clock: The mitochondria?. J. Am. Geriatr. Soc.

[16] Dei R., Takeda A., Niwa H., Li M., Nakagomi Y., Watanabe M., Inagaki T., Washimi Y., Yasuda Y., Horie K. (2002). Lipid peroxidation and advanced glycation end products in the brain in normal aging and in Alzheimer’s disease. Acta Neuropathol.

[17] Lovell M.A., Ehmann W.D., Mattson M.P., Markesbery W.R. (1997). Elevated 4-hydroxynonenal in ventricular fluid in Alzheimer’s disease. Neurobiol. Aging.

[18] Starke-Reed P.E., Oliver C.N. (1989). Protein oxidation and proteolysis during aging and oxidative stress. Arch. Biochem. Biophys.

[19] Parkes T.L., Elia A.J., Dickinson D., Hilliker A.J., Phillips J.P., Boulianne G.L. (1998). Extension of Drosophila lifespan by overexpression of human SOD1 in motorneurons. Nat. Genet.

[20] Magwere T., West M., Riyahi K., Murphy M.P., Smith R.A., Partridge L. (2006). The effects of exogenous antioxidants on lifespan and oxidative stress resistance in Drosophila melanogaster. Mech. Ageing Dev.

[21] Sohal R.S. (2002). Role of oxidative stress and protein oxidation in the aging process. Free Radic. Biol. Med.

[22] Muller F.L., Lustgarten M.S., Jang Y., Richardson A., van Remmen H. (2007). Trends in oxidative aging theories. Free Radic. Biol. Med.

[23] Przedborski S., Ischiropoulos H. (2005). Reactive oxygen and nitrogen species: Weapons of neuronal destruction in models of Parkinson’s disease. Antioxid. Redox Signal.

[24] Schapira A.H. (1997). Pathogenesis of Parkinson’s disease. Baillieres Clin. Neurol.

[25] Alam Z.I., Daniel S.E., Lees A.J., Marsden D.C., Jenner P., Halliwell B. (1997). A generalised increase in protein carbonyls in the brain in Parkinson’s but not incidental Lewy body disease. J. Neurochem.

[26] Dexter D., Carter C., Agid F., Agid Y., Lees A.J., Jenner P., Marsden C.D. (1986). Lipid peroxidation as cause of nigral cell death in Parkinson’s disease. Lancet.

[27] Hamilton M.L., van Remmen H., Drake J.A., Yang H., Guo Z.M., Kewitt K., Walter C.A., Richardson A. (2001). Does oxidative damage to DNA increase with age?. Proc. Natl. Acad. Sci. USA.

[28] Oliver C.N., Ahn B.W., Moerman E.J., Goldstein S., Stadtman E.R. (1987). Age-related changes in oxidized proteins. J. Biol. Chem.

[29] Calne D.B., Langston J.W. (1983). Aetiology of Parkinson’s disease. Lancet.

[30] Morens D.M., Davis J.W., Grandinetti A., Ross G.W., Popper J.S., White L.R. (1996). Epidemiologic observations on Parkinson’s disease: Incidence and mortality in a prospective study of middle-aged men. Neurology.

[31] De Lau L.M., Giesbergen P.C., de Rijk M.C., Hofman A., Koudstaal P.J., Breteler M.M. (2004). Incidence of parkinsonism and Parkinson disease in a general population: The Rotterdam Study. Neurology.

[32] De Rijk M.C., Tzourio C., Breteler M.M., Dartigues J.F., Amaducci L., Lopez-Pousa S., Manubens-Bertran J.M., Alperovitch A., Rocca W.A. (1997). Prevalence of parkinsonism and Parkinson’s disease in Europe: The Europarkinson collaborative study. European Community Concerted Action on the Epidemiology of Parkinson’s disease. J. Neurol. Neurosurg. Psychiatry.

[33] Fearnley J.M., Lees A.J. (1991). Ageing and Parkinson’s disease: Substantia nigra regional selectivity. Brain.

[34] Mann D.M., Yates P.O. (1983). Possible role of neuromelanin in the pathogenesis of Parkinson’s disease. Mech. Ageing Dev.

[35] Dorsey E.R., Constantinescu R., Thompson J.P., Biglan K.M., Holloway R.G., Kieburtz K., Marshall F.J., Ravina B.M., Schifitto G., Siderowf A. (2007). Projected number of people with Parkinson disease in the most populous nations, 2005 through 2030. Neurology.

[36] Marsden C.D. (1990). Parkinson’s disease. Lancet.

[37] Beal M.F. (2009). Therapeutic approaches to mitochondrial dysfunction in Parkinson’s disease. Parkinsonism Relat. Disord.

[38] Kushnareva Y., Murphy A.N., Andreyev A. (2002). Complex I-mediated reactive oxygen species generation: Modulation by cytochrome c and NAD(P)^+^ oxidation-reduction state. Biochem. J.

[39] Fasano M., Bergamasco B., Lopiano L. (2006). Modifications of the iron-neuromelanin system in Parkinson’s disease. J. Neurochem.

[40] Ischiropoulos H., Beckman J.S. (2003). Oxidative stress and nitration in neurodegeneration: Cause, effect, or association?. J. Clin. Invest.

[41] Linnane A.W., Marzuki S., Ozawa T., Tanaka M. (1989). Mitochondrial DNA mutations as an important contributor to ageing and degenerative diseases. Lancet.

[42] Kraytsberg Y., Kudryavtseva E., McKee A.C., Geula C., Kowall N.W., Khrapko K. (2006). Mitochondrial DNA deletions are abundant and cause functional impairment in aged human substantia nigra neurons. Nat. Genet.

[43] Bender A., Krishnan K.J., Morris C.M., Taylor G.A., Reeve A.K., Perry R.H., Jaros E., Hersheson J.S., Betts J., Klopstock T. (2006). High levels of mitochondrial DNA deletions in substantia nigra neurons in aging and Parkinson disease. Nat. Genet.

[44] Anderson S., Bankier A.T., Barrell B.G., de Bruijn M.H., Coulson A.R., Drouin J., Eperon I.C., Nierlich D.P., Roe B.A., Sanger F. (1981). Sequence and organization of the human mitochondrial genome. Nature.

[45] Pinton P., Rimessi A., Marchi S., Orsini F., Migliaccio E., Giorgio M., Contursi C., Minucci S., Mantovani F., Wieckowski M.R. (2007). Protein kinase C beta and prolyl isomerase 1 regulate mitochondrial effects of the life-span determinant p66Shc. Science.

[46] Ruan H., Tang X.D., Chen M.L., Joiner M.L., Sun G., Brot N., Weissbach H., Heinemann S.H., Iverson L., Wu C.F. (2002). High-quality life extension by the enzyme peptide methionine sulfoxide reductase. Proc. Natl. Acad. Sci. USA.

[47] Kujoth G.C., Hiona A., Pugh T.D., Someya S., Panzer K., Wohlgemuth S.E., Hofer T., Seo A.Y., Sullivan R., Jobling W.A. (2005). Mitochondrial DNA mutations, oxidative stress, and apoptosis in mammalian aging. Science.

[48] Trifunovic A., Wredenberg A., Falkenberg M., Spelbrink J.N., Rovio A.T., Bruder C.E., Bohlooly Y.M., Gidlof S., Oldfors A., Wibom R. (2004). Premature ageing in mice expressing defective mitochondrial DNA polymerase. Nature.

[49] Davidzon G., Greene P., Mancuso M., Klos K.J., Ahlskog J.E., Hirano M., DiMauro S. (2006). Early-onset familial parkinsonism due to POLG mutations. Ann. Neurol.

[50] Ahluwalia N., Gordon M.A., Handte G., Mahlon M., Li N.Q., Beard J.L., Weinstock D., Ross A.C. (2000). Iron status and stores decline with age in Lewis rats. J. Nutr.

[51] Bonifati V., Rizzu P., van Baren M.J., Schaap O., Breedveld G.J., Krieger E., Dekker M.C., Squitieri F., Ibanez P., Joosse M. (2003). Mutations in the DJ-1 gene associated with autosomal recessive early-onset parkinsonism. Science.

[52] Van Duijn C.M., Dekker M.C., Bonifati V., Galjaard R.J., Houwing-Duistermaat J.J., Snijders P.J., Testers L., Breedveld G.J., Horstink M., Sandkuijl L.A. (2001). Park7, a novel locus for autosomal recessive early-onset parkinsonism, on chromosome 1p36. Am. J. Hum. Genet.

[53] Lesage S., Brice A. (2012). Role of Mendelian genes in “sporadic” Parkinson’s disease. Parkinsonism Relat. Disord.

[54] Horowitz M.P., Greenamyre J.T. (2010). Gene-environment interactions in Parkinson’s disease: The importance of animal modeling. Clin. Pharmacol. Ther.

[55] Meulener M.C., Xu K., Thomson L., Ischiropoulos H., Bonini N.M. (2006). Mutational analysis of DJ-1 in Drosophila implicates functional inactivation by oxidative damage and aging. Proc. Natl. Acad. Sci. USA.

[56] Yamaguchi H., Shen J (2007). Absence of dopaminergic neuronal degeneration and oxidative damage in aged DJ-1-deficient mice. Mol. Neurodegener.

[57] Kawajiri S., Saiki S., Sato S., Hattori N. (2011). Genetic mutations and functions of PINK1. Trends Pharmacol. Sci.

[58] Moore D.J., West A.B., Dawson V.L., Dawson T.M. (2005). Molecular pathophysiology of Parkinson’s disease. Annu. Rev. Neurosci.

[59] Todd A.M., Staveley B.E. (2008). Pink1 suppresses alpha-synuclein-induced phenotypes in a Drosophila model of Parkinson’s disease. Genome.

[60] Haywood A.F., Staveley B.E. (2004). Parkin counteracts symptoms in a Drosophila model of Parkinson’s disease. BMC Neurosci.

[61] Palacino J.J., Sagi D., Goldberg M.S., Krauss S., Motz C., Wacker M., Klose J., Shen J. (2004). Mitochondrial dysfunction and oxidative damage in parkin-deficient mice. J. Biol. Chem.

[62] Perez F.A., Palmiter R.D. (2005). Parkin-deficient mice are not a robust model of parkinsonism. Proc. Natl. Acad. Sci. USA.

[63] Periquet M., Corti O., Jacquier S., Brice A. (2005). Proteomic analysis of parkin knockout mice: Alterations in energy metabolism, protein handling and synaptic function. J. Neurochem.

[64] Kitada T., Asakawa S., Hattori N., Matsumine H., Yamamura Y., Minoshima S., Yokochi M., Mizuno Y., Shimizu N. (1998). Mutations in the parkin gene cause autosomal recessive juvenile parkinsonism. Nature.

[65] Hedrich K., Eskelson C., Wilmot B., Marder K., Harris J., Garrels J., Meija-Santana H., Vieregge P., Jacobs H., Bressman S.B. (2004). Distribution, type, and origin of Parkin mutations: Review and case studies. Mov. Disord.

[66] Lucking C.B., Durr A., Bonifati V., Vaughan J., de Michele G., Gasser T., Harhangi B.S., Meco G., Denefle P., Wood N.W. (2000). Association between early-onset Parkinson’s disease and mutations in the parkin gene. N. Engl. J. Med.

[67] Periquet M., Latouche M., Lohmann E., Rawal N., de Michele G., Ricard S., Teive H., Fraix V., Vidailhet M., Nicholl D. (2003). Parkin mutations are frequent in patients with isolated early-onset parkinsonism. Brain.

[68] Lohmann E., Periquet M., Bonifati V., Wood N.W., de Michele G., Bonnet A.M., Fraix V., Broussolle E., Horstink M.W., Vidailhet M. (2003). How much phenotypic variation can be attributed to parkin genotype?. Ann. Neurol.

[69] Foroud T., Uniacke S.K., Liu L., Pankratz N., Rudolph A., Halter C., Shults C., Marder K., Conneally P.M., Nichols W.C. (2003). Heterozygosity for a mutation in the parkin gene leads to later onset Parkinson disease. Neurology.

[70] Sun M., Latourelle J.C., Wooten G.F., Lew M.F., Klein C., Shill H.A., Golbe L.I., Mark M.H., Racette B.A., Perlmutter J.S. (2006). Influence of heterozygosity for parkin mutation on onset age in familial Parkinson disease: The GenePD study. Arch. Neurol.

[71] Broussolle E., Lucking C.B., Ginovart N., Pollak P., Remy P., Durr A. (2000). [18 F]-dopa PET study in patients with juvenile-onset PD and parkin gene mutations. Neurology.

[72] Shulman J.M., de Jager P.L., Feany M.B. (2011). Parkinson’s disease: Genetics and pathogenesis. Annu. Rev. Pathol.

[73] Slivka A., Cohen G. (1985). Hydroxyl radical attack on dopamine. J. Biol. Chem.

[74] Fornstedt B., Pileblad E., Carlsson A. (1990). *In vivo* autoxidation of dopamine in guinea pig striatum increases with age. J. Neurochem.

[75] Sulzer D., Zecca L. (2000). Intraneuronal dopamine-quinone synthesis: A review. Neurotox. Res.

[76] Mann J.J., Stanley M. (1984). Postmortem monoamine oxidase enzyme kinetics in the frontal cortex of suicide victims and controls. Acta Psychiatr. Scand.

[77] Jossan S.S., Gillberg P.G., d’Argy R., Aquilonius S.M., Langstrom B., Halldin C., Oreland L. (1991). Quantitative localization of human brain monoamine oxidase B by large section autoradiography using l-[3H]deprenyl. Brain Res.

[78] Strolin Benedetti M., Dostert P. (1989). Monoamine oxidase, brain ageing and degenerative diseases. Biochem. Pharmacol.

[79] Thomas B.B.M. (2007). Parkinson’s disease. Hum. Mol. Genet.

[80] Morgan D.G., May P.C., Finch C.E. (1987). Dopamine and serotonin systems in human and rodent brain: Effects of age and neurodegenerative disease. J. Am. Geriatr. Soc.

[81] Kish S.J., Shannak K., Rajput A., Deck J.H., Hornykiewicz O. (1992). Aging produces a specific pattern of striatal dopamine loss: Implications for the etiology of idiopathic Parkinson’s disease. J. Neurochem.

[82] Thannickal T.C., Lai Y.Y., Siegel J.M. (2008). Hypocretin (orexin) and melanin concentrating hormone loss and the symptoms of Parkinson’s disease. Brain.

[83] Cruz-Muros I., Afonso-Oramas D., Abreu P., Barroso-Chinea P., Rodriguez M., Gonzalez M.C., Hernandez T.G. (2007). Aging of the rat mesostriatal system: Differences between the nigrostriatal and the mesolimbic compartments. Exp. Neurol.

[84] Goudsmit E., Feenstra M.G., Swaab D.F. (1990). Central monoamine metabolism in the male Brown-Norway rat in relation to aging and testosterone. Brain Res. Bull.

[85] Volkow N.D., Ding Y.S., Fowler J.S., Wang G.J., Logan J., Gatley S.J., Hitzemann R., Smith G., Fields S.D., Gur R. (1996). Dopamine transporters decrease with age. J. Nucl. Med.

[86] Caudle W.M., Richardson J.R., Wang M.Z., Taylor T.N., Guillot T.S., McCormack A.L., Colebrooke R.E., di Monte D.A., Emson P.C., Miller G.W. (2007). Reduced vesicular storage of dopamine causes progressive nigrostriatal neurodegeneration. J. Neurosci.

[87] Gonzalez-Hernandez T., Barroso-Chinea P., de La Cruz Muros I., del Mar Perez-Delgado M., Rodriguez M. (2004). Expression of dopamine and vesicular monoamine transporters and differential vulnerability of mesostriatal dopaminergic neurons. J. Comp. Neurol.

[88] Cantuti-Castelvetri I., Shukitt-Hale B., Joseph J.A. (2003). Dopamine neurotoxicity: Age-dependent behavioral and histological effects. Neurobiol. Aging.

[89] De Keyser J., Ebinger G., Vauquelin G. (1990). Age-related changes in the human nigrostriatal dopaminergic system. Ann. Neurol.

[90] Irwin I., DeLanney L.E., McNeill T., Chan P., Forno L.S., Murphy G.M., di Monte D.A., Sandy M.S., Langston J.W. (1994). Aging and the nigrostriatal dopamine system: A non-human primate study. Neurodegeneration.

[91] Borges C.R., Geddes T., Watson J.T., Kuhn D.M. (2002). Dopamine biosynthesis is regulated by *S*-glutathionylation. Potential mechanism of tyrosine hydroxylast inhibition during oxidative stress. J. Biol. Chem.

[92] De La Cruz C.P., Revilla E., Venero J.L., Ayala A., Cano J., Machado A. (1996). Oxidative inactivation of tyrosine hydroxylase in substantia nigra of aged rat. Free Radic. Biol. Med.

[93] Ischiropoulos H. (1998). Biological tyrosine nitration: A pathophysiological function of nitric oxide and reactive oxygen species. Arch. Biochem. Biophys.

[94] Goedert M. (1997). Familial Parkinson’s disease. The awakening of alpha-synuclein. Nature.

[95] Yu S., Ueda K., Chan P. (2005). Alpha-synuclein and dopamine metabolism. Mol. Neurobiol.

[96] Perez R.G., Hastings T.G. (2004). Could a loss of alpha-synuclein function put dopaminergic neurons at risk?. J. Neurochem.

[97] Kim K.S., Choi S.Y., Kwon H.Y., Won M.H., Kang T.C., Kang J.H. (2002). Aggregation of alpha-synuclein induced by the Cu,Zn-superoxide dismutase and hydrogen peroxide system. Free Radic. Biol. Med.

[98] Conway K.A., Rochet J.C., Bieganski R.M., Lansbury P.T. (2001). Kinetic stabilization of the alpha-synuclein protofibril by a dopamine-alpha-synuclein adduct. Science.

[99] Xu J., Kao S.Y., Lee F.J., Song W., Jin L.W., Yankner B.A. (2002). Dopamine-dependent neurotoxicity of alpha-synuclein: A mechanism for selective neurodegeneration in Parkinson disease. Nat. Med.

[100] Borghi R., Marchese R., Negro A., Marinelli L., Forloni G., Zaccheo D., Abbruzzese G., Tabaton M. (2000). Full length alpha-synuclein is present in cerebrospinal fluid from Parkinson’s disease and normal subjects. Neurosci. Lett.

[101] Chu Y., Kordower J.H. (2007). Age-associated increases of alpha-synuclein in monkeys and humans are associated with nigrostriatal dopamine depletion: Is this the target for Parkinson’s disease?. Neurobiol. Dis.

[102] Jellinger K.A. (2004). Lewy body-related alpha-synucleinopathy in the aged human brain. J. Neural. Transm.

[103] Li W., Lesuisse C., Xu Y., Troncoso J.C., Price D.L., Lee M.K. (2004). Stabilization of alpha-synuclein protein with aging and familial parkinson’s disease-linked A53T mutation. J. Neurosci.

[104] Buchman A.S., Shulman J.M., Nag S., Leurgans S.E., Arnold S.E., Morris M.C., Schneider J.A., Bennett D.A. (2012). Nigral pathology and parkinsonian signs in elders without Parkinson disease. Ann. Neurol.

[105] Adamczyk A., Kazmierczak A., Strosznajder J.B. (2006). Alpha-synuclein and its neurotoxic fragment inhibit dopamine uptake into rat striatal synaptosomes. Relationship to nitric oxide. Neurochem. Int.

[106] Saito Y., Kawashima A., Ruberu N.N., Fujiwara H., Koyama S., Sawabe M., Arai T., Nagura H., Yamanouchi H., Hasegawa M. (2003). Accumulation of phosphorylated alpha-synuclein in aging human brain. J. Neuropathol. Exp. Neurol.

[107] Junn E., Mouradian M.M. (2002). Human alpha-synuclein over-expression increases intracellular reactive oxygen species levels and susceptibility to dopamine. Neurosci. Lett.

[108] Hashimoto M., Hsu L.J., Xia Y., Takeda A., Sisk A., Sundsmo M., Masliah E. (1999). Oxidative stress induces amyloid-like aggregate formation of NACP/alpha-synuclein *in vitro*. Neuroreport.

[109] Turnbull S., Tabner B.J., El-Agnaf O.M., Moore S., Davies Y., Allsop D. (2001). Alpha-Synuclein implicated in Parkinson’s disease catalyses the formation of hydrogen peroxide *in vitro*. Free Radic. Biol. Med.

[110] Hartl F.U. (1996). Molecular chaperones in cellular protein folding. Nature.

[111] Neumann M., Kahle P.J., Giasson B.I., Ozmen L., Borroni E., Spooren W., Muller V., Odoy S., Fujiwara H., Hasegawa M. (2002). Misfolded proteinase K-resistant hyperphosphorylated alpha-synuclein in aged transgenic mice with locomotor deterioration and in human alpha-synucleinopathies. J. Clin. Invest.

[112] Beach T.G., Walker D.G., Sue L.I., Newell A., Adler C.C., Joyce J.N. (2004). Substantia nigra Marinesco bodies are associated with decreased striatal expression of dopaminergic markers. J. Neuropathol. Exp. Neurol.

[113] Braak H., del Tredici K., Rub U., de Vos R.A., Jansen Steur E.N., Braak E. (2003). Staging of brain pathology related to sporadic Parkinson’s disease. Neurobiol. Aging.

[114] Cuervo A.M., Stefanis L., Fredenburg R., Lansbury P.T., Sulzer D. (2004). Impaired degradation of mutant alpha-synuclein by chaperone-mediated autophagy. Science.

[115] Lee H.J., Khoshaghideh F., Patel S., Lee S.J. (2004). Clearance of alpha-synuclein oligomeric intermediates via the lysosomal degradation pathway. J. Neurosci.

[116] Webb J.L., Ravikumar B., Atkins J., Skepper J.N., Rubinsztein D.C. (2003). Alpha-Synuclein is degraded by both autophagy and the proteasome. J. Biol. Chem.

[117] Perry V.H., Nicoll J.A., Holmes C. (2010). Microglia in neurodegenerative disease. Nat. Rev. Neurol.

[118] Xu H., Chen M., Forrester J.V. (2009). Para-inflammation in the aging retina. Prog. Retin. Eye Res.

[119] Price N.E., Wadzinski B., Mumby M.C. (1999). An anchoring factor targets protein phosphatase 2A to brain microtubules. Brain Res. Mol. Brain Res.

[120] Klegeris A., McGeer E.G., McGeer P.L. (2007). Therapeutic approaches to inflammation in neurodegenerative disease. Curr. Opin. Neurol.

[121] Beach T.G., Sue L.I., Walker D.G., Lue L.F., Connor D.J., Caviness J.N., Sabbagh M.N., Adler C.H. (2007). Marked microglial reaction in normal aging human substantia nigra: Correlation with extraneuronal neuromelanin pigment deposits. Acta Neuropathol.

[122] Roy A., Jana A., Yatish K., Freidt M.B., Fung Y.K., Martinson J.A., Pahan K. (2008). Reactive oxygen species up-regulate CD11b in microglia via nitric oxide: Implications for neurodegenerative diseases. Free Radic. Biol. Med.

[123] McGeer P.L., Itagaki S., Boyes B.E., McGeer E.G. (1988). Reactive microglia are positive for HLA-DR in the substantia nigra of Parkinson’s and Alzheimer’s disease brains. Neurology.

[124] Imamura K., Hishikawa N., Sawada M., Nagatsu T., Yoshida M., Hashizume Y. (2003). Distribution of major histocompatibility complex class II-positive microglia and cytokine profile of Parkinson’s disease brains. Acta Neuropathol.

[125] Gerhard A., Pavese N., Hotton G., Turkheimer F., Es M., Hammers A., Eggert K., Oertel W., Banati R.B., Brooks D.J. (2006). *In vivo* imaging of microglial activation with [11C](R)-PK11195 PET in idiopathic Parkinson’s disease. Neurobiol. Dis.

[126] Aloisi F, Kettenmann H., Ransom B.R. (2005). Cytokine Production. Neuroglia.

[127] Sierra A., Gottfried-Blackmore A.C., McEwen B.S., Bulloch K. (2007). Microglia derived from aging mice exhibit an altered inflammatory profile. Glia.

[128] Sawada M., Sawada H., Nagatsu T. (2008). Effects of aging on neuroprotective and neurotoxic properties of microglia in neurodegenerative diseases. Neurodegener. Dis.

[129] Godbout J.P., Chen J., Abraham J., Richwine A.F., Berg B.M., Kelley K.W., Johnson R.W. (2005). Exaggerated neuroinflammation and sickness behavior in aged mice following activation of the peripheral innate immune system. FASEB J.

[130] Damani M.R., Zhao L., Fontainhas A.M., Amaral J., Fariss R.N., Wong W.T. (2011). Age-related alterations in the dynamic behavior of microglia. Aging Cell.

[131] Gregory A., Polster B.J., Hayflick S.J. (2009). Clinical and genetic delineation of neurodegeneration with brain iron accumulation. J. Med. Genet.

[132] Colton C.A., Gilbert D.L. (1987). Production of superoxide anions by a CNS macrophage, the microglia. FEBS Lett.

[133] Biemond P., van Eijk H.G., Swaak A.J., Koster J.F. (1984). Iron mobilization from ferritin by superoxide derived from stimulated polymorphonuclear leukocytes. Possible mechanism in inflammation diseases. J. Clin. Invest.

[134] Agrawal R., Sharma P.K., Rao G.S. (2001). Release of iron from ferritin by metabolites of benzene and superoxide radical generating agents. Toxicology.

[135] Tanaka M., Sotomatsu A., Yoshida T., Hirai S., Nishida A. (1994). Detection of superoxide production by activated microglia using a sensitive and specific chemiluminescence assay and microglia-mediated PC12h cell death. J. Neurochem.

[136] Chen Y., Swanson R.A. (2003). Astrocytes and brain injury. J. Cereb. Blood Flow Metab.

[137] Nichols N.R. (1999). Glial responses to steroids as markers of brain aging. J. Neurobiol.

[138] Porchet R., Probst A., Bouras C., Draberova E., Draber P., Riederer B.M. (2003). Analysis of glial acidic fibrillary protein in the human entorhinal cortex during aging and in Alzheimer’s disease. Proteomics.

[139] Peinado M.A., Quesada A., Pedrosa J.A., Torres M.I., Martinez M., Esteban F.J., del Moral M.L., Hernandez R., Rodrigo J., Peinado J.M. (1998). Quantitative and ultrastructural changes in glia and pericytes in the parietal cortex of the aging rat. Microsc. Res. Tech.

[140] Pilegaard K., Ladefoged O. (1996). Total number of astrocytes in the molecular layer of the dentate gyrus of rats at different ages. Anal. Quant. Cytol. Histol.

[141] Rozovsky I., Finch C.E., Morgan T.E. (1998). Age-related activation of microglia and astrocytes: *In vitro* studies show persistent phenotypes of aging, increased proliferation, and resistance to down-regulation. Neurobiol. Aging.

[142] Ballatori N., Krance S.M., Notenboom S., Shi S., Tieu K., Hammond C.L. (2009). Glutathione dysregulation and the etiology and progression of human diseases. Biol. Chem.

[143] Rice M.E., Russo-Menna I. (1998). Differential compartmentalization of brain ascorbate and glutathione between neurons and glia. Neuroscience.

[144] Gegg M.E., Beltran B., Salas-Pino S., Bolanos J.P., Clark J.B., Moncada S., Heales S.J. (2003). Differential effect of nitric oxide on glutathione metabolism and mitochondrial function in astrocytes and neurones: Implications for neuroprotection/neurodegeneration?. J. Neurochem.

[145] Sian J., Dexter D.T., Lees A.J., Daniel S., Agid Y., Javoy-Agid F., Jenner P., Marsden C.D. (1994). Alterations in glutathione levels in Parkinson’s disease and other neurodegenerative disorders affecting basal ganglia. Ann. Neurol.

[146] O’Callaghan J.P., Miller D.B. (1991). The concentration of glial fibrillary acidic protein increases with age in the mouse and rat brain. Neurobiol. Aging.

[147] Uchida K., Kihara N., Hashimoto K., Nakayama H., Yamaguchi R., Tateyama S. (2003). Age-related histological changes in the canine substantia nigra. J. Vet. Med. Sci.

[148] Gu X.L., Long C.X., Sun L., Xie C., Lin X., Cai H (2010). Astrocytic expression of Parkinson’s disease-related A53T alpha-synuclein causes neurodegeneration in mice. Mol. Brain.

[149] Giasson B.I., Duda J.E., Quinn S.M., Zhang B., Trojanowski J.Q., Lee V.M. (2002). Neuronal alpha-synucleinopathy with severe movement disorder in mice expressing A53T human alpha-synuclein. Neuron.

[150] Lee M.K., Stirling W., Xu Y., Xu X., Qui D., Mandir A.S., Dawson T.M., Copeland N.G., Jenkins N.A., Price D.L. (2002). Human alpha-synuclein-harboring familial Parkinson’s disease-linked Ala-53 → Thr mutation causes neurodegenerative disease with alpha-synuclein aggregation in transgenic mice. Proc. Natl. Acad. Sci. USA.

[151] Park S.J., Lee J.H., Kim H.Y., Choi Y.H., Park J.S., Suh Y.H., Park S.M., Joe E.H., Jou I. (2012). Astrocytes, but not microglia, rapidly sense H_2_O_2_ via STAT6 phosphorylation, resulting in cyclooxygenase-2 expression and prostaglandin release. J. Immunol.

[152] Khachaturian Z.S. (1989). The role of calcium regulation in brain aging: Reexamination of a hypothesis. Aging (Milano).

[153] Bean B.P. (2007). The action potential in mammalian central neurons. Nat. Rev. Neurosci.

[154] Bonci A., Grillner P., Mercuri N.B., Bernardi G. (1998). L-Type calcium channels mediate a slow excitatory synaptic transmission in rat midbrain dopaminergic neurons. J. Neurosci.

[155] Puopolo M., Raviola E., Bean B.P. (2007). Roles of subthreshold calcium current and sodium current in spontaneous firing of mouse midbrain dopamine neurons. J. Neurosci.

[156] Chan C.S., Guzman J.N., Ilijic E., Mercer J.N., Rick C., Tkatch T., Meredith G.E., Surmeier D.J. (2007). “Rejuvenation” protects neurons in mouse models of Parkinson’s disease”. Nature.

[157] Pignatelli A., Kobayashi K., Okano H., Belluzzi O. (2005). Functional properties of dopaminergic neurones in the mouse olfactory bulb. J. Physiol.

[158] German D.C., Manaye K.F., Sonsalla P.K., Brooks B.A. (1992). Midbrain dopaminergic cell loss in Parkinson’s disease and MPTP-induced parkinsonism: Sparing of calbindin-D28k-containing cells. Ann. N. Y. Acad. Sci.

[159] Belzunegui S., San Sebastian W., Garrido-Gil P., Izal-Azcarate A., Vazquez-Claverie M., Lopez B., Marcilla I., Lanciego J.L., Luquin M.R. (2007). The number of dopaminergic cells is increased in the olfactory bulb of monkeys chronically exposed to MPTP. Synapse.

[160] Rizzuto R., Pozzan T. (2006). Microdomains of intracellular Ca^2+^: Molecular determinants and functional consequences. Physiol. Rev.

[161] Verkhratsky A. (2005). Physiology and pathophysiology of the calcium store in the endoplasmic reticulum of neurons. Physiol. Rev.

[162] Roveri A., Coassin M., Maiorino M., Zamburlini A., van Amsterdam F.T., Ratti E., Ursini F. (1992). Effect of hydrogen peroxide on calcium homeostasis in smooth muscle cells. Arch. Biochem. Biophys.

[163] Wang H., Joseph J.A. (2000). Mechanisms of hydrogen peroxide-induced calcium dysregulation in PC12 cells. Free Radic. Biol. Med.

[164] Thibault O., Landfield P.W. (1996). Increase in single L-type calcium channels in hippocampal neurons during aging. Science.

[165] Altamura S., Muckenthaler M.U. (2009). Iron toxicity in diseases of aging: Alzheimer’s disease, Parkinson’s disease and atherosclerosis. J. Alzheimers Dis.

[166] Halliwell B., Gutteridge J.M. (1984). Oxygen toxicity, oxygen radicals, transition metals and disease. Biochem. J.

[167] Dexter D.T., Sian J., Jenner P., Marsden C.D. (1993). Implications of alterations in trace element levels in brain in Parkinson’s disease and other neurological disorders affecting the basal ganglia. Adv. Neurol.

[168] Connor J.R., Menzies S.L., St Martin S.M., Mufson E.J. (1990). Cellular distribution of transferrin, ferritin, and iron in normal and aged human brains. J. Neurosci. Res.

[169] Roskams A.J., Connor J.R. (1994). Iron, transferrin, and ferritin in the rat brain during development and aging. J. Neurochem.

[170] Bartzokis G., Tishler T.A., Lu P.H., Villablanca P., Altshuler L.L., Carter M., Huang D., Edwards N., Mintz J. (2007). Brain ferritin iron may influence age- and gender-related risks of neurodegeneration. Neurobiol. Aging.

[171] Berg D., Youdim M.B., Riederer P. (2004). Redox imbalance. Cell Tissue Res.

[172] Seo A.Y., Xu J., Servais S., Hofer T., Marzetti E., Wohlgemuth S.E., Knutson M.D., Chung H.Y., Leeuwenburgh C. (2008). Mitochondrial iron accumulation with age and functional consequences. Aging Cell.

[173] Aquino D., Bizzi A., Grisoli M., Garavaglia B., Bruzzone M.G., Nardocci N., Savoiardo M., Chiapparini L. (2009). Age-related iron deposition in the basal ganglia: Quantitative analysis in healthy subjects. Radiology.

[174] Halliwell B., Gutteridge J.M. (1986). Oxygen free radicals and iron in relation to biology and medicine: Some problems and concepts. Arch. Biochem. Biophys.

[175] Martin W.R., Ye F.Q., Allen P.S. (1998). Increasing striatal iron content associated with normal aging. Mov. Disord.

[176] Zecca L., Stroppolo A., Gatti A., Tampellini D., Toscani M., Gallorini M., Giaveri G., Arosio P., Santambrogio P., Fariello R.G. (2004). The role of iron and copper molecules in the neuronal vulnerability of locus coeruleus and substantia nigra during aging. Proc. Natl. Acad. Sci. USA.

[177] Leibold E.A., Gahring L.C., Rogers S.W. (2001). Immunolocalization of iron regulatory protein expression in the murine central nervous system. Histochem. Cell Biol.

[178] Han J., Cheng F.C., Yang Z., Dryhurst G. (1999). Inhibitors of mitochondrial respiration, iron (II), and hydroxyl radical evoke release and extracellular hydrolysis of glutathione in rat striatum and substantia nigra: Potential implications to Parkinson’s disease. J. Neurochem.

[179] Lan J., Jiang D.H. (1997). Desferrioxamine and vitamin E protect against iron and MPTP-induced neurodegeneration in mice. J. Neural. Transm.

[180] Shachar D.B., Kahana N., Kampel V., Warshawsky A., Youdim M.B. (2004). Neuroprotection by a novel brain permeable iron chelator, VK-28, against 6-hydroxydopamine lession in rats. Neuropharmacology.

[181] Nguyen T., Sherratt P.J., Nioi P., Yang C.S., Pickett C.B. (2005). Nrf2 controls constitutive and inducible expression of ARE-driven genes through a dynamic pathway involving nucleocytoplasmic shuttling by Keap1. J. Biol. Chem.

[182] Moi P., Chan K., Asunis I., Cao A., Kan Y.W. (1994). Isolation of NF-E2-related factor 2 (Nrf2), a NF-E2-like basic leucine zipper transcriptional activator that binds to the tandem NF-E2/AP1 repeat of the beta-globin locus control region. Proc. Natl. Acad. Sci. USA.

[183] Ramsey C.P., Glass C.A., Montgomery M.B., Lindl K.A., Ritson G.P., Chia L.A., Hamilton R.L., Chu C.T., Jordan-Sciutto K.L. (2007). Expression of Nrf2 in neurodegenerative diseases. J. Neuropathol. Exp. Neurol.

[184] Von Otter M., Landgren S., Nilsson S., Celojevic D., Bergstrom P., Hakansson A., Nissbrandt H., Drozdzik M., Bialecka M., Kurzawski M. (2010). Association of Nrf2-encoding NFE2L2 haplotypes with Parkinson’s disease. BMC Med. Genet.

[185] Suh J.H., Shenvi S.V., Dixon B.M., Liu H., Jaiswal A.K., Liu R.M., Hagen T.M. (2004). Decline in transcriptional activity of Nrf2 causes age-related loss of glutathione synthesis, which is reversible with lipoic acid. Proc. Natl. Acad. Sci. USA.

[186] Rojo A.I., Sagarra M.R., Cuadrado A. (2008). GSK-3beta down-regulates the transcription factor Nrf2 after oxidant damage: Relevance to exposure of neuronal cells to oxidative stress. J. Neurochem.

[187] Ikeyama S., Kokkonen G., Shack S., Wang X.T., Holbrook N.J. (2002). Loss in oxidative stress tolerance with aging linked to reduced extracellular signal-regulated kinase and Akt kinase activities. FASEB J.

[188] Kozikowski A.P., Gaisina I.N., Petukhov P.A., Sridhar J., King L.T., Blond S.Y., Duka T., Rusnak M., Sidhu A. (2006). Highly potent and specific GSK-3beta inhibitors that block tau phosphorylation and decrease alpha-synuclein protein expression in a cellular model of Parkinson’s disease. Chem. Med. Chem.

[189] Chen G., Bower K.A., Ma C., Fang S., Thiele C.J., Luo J. (2004). Glycogen synthase kinase 3beta (GSK3beta) mediates 6-hydroxydopamine-induced neuronal death. FASEB J.

[190] Chen C., Pung D., Leong V., Hebbar V., Shen G., Nair S., Li W., Kong A.N. (2004). Induction of detoxifying enzymes by garlic organosulfur compounds through transcription factor Nrf2: Effect of chemical structure and stress signals. Free Radic. Biol. Med.

[191] Silva R.M., Kuan C.Y., Rakic P., Burke R.E. (2005). Mixed lineage kinase-c-jun *N*-terminal kinase signaling pathway: A new therapeutic target in Parkinson’s disease. Mov. Disord.

[192] Dagda R.K., Zhu J., Chu C.T. (2009). Mitochondrial kinases in Parkinson’s disease: Converging insights from neurotoxin and genetic models. Mitochondrion.

[193] Shih P.H., Yen G.C. (2007). Differential expressions of antioxidant status in aging rats: The role of transcriptional factor Nrf2 and MAPK signaling pathway. Biogerontology.

[194] Hsieh C.C., Rosenblatt J.I., Papaconstantinou J. (2003). Age-associated changes in SAPK/JNK and p38 MAPK signaling in response to the generation of ROS by 3-nitropropionic acid. Mech. Ageing Dev.

[195] Hsieh C.C., Papaconstantinou J. (2009). Dermal fibroblasts from long-lived Ames dwarf mice maintain their *in vivo* resistance to mitochondrial generated reactive oxygen species (ROS). Aging (Albany NY).

[196] Helenius M., Hanninen M., Lehtinen S.K., Salminen A. (1996). Changes associated with aging and replicative senescence in the regulation of transcription factor nuclear factor-kappa B. Biochem. J.

[197] Korhonen P., Helenius M., Salminen A. (1997). Age-related changes in the regulation of transcription factor NF-kappa B in rat brain. Neurosci. Lett.

[198] Dehmer T., Lindenau J., Haid S., Dichgans J., Schulz J.B. (2000). Deficiency of inducible nitric oxide synthase protects against MPTP toxicity *in vivo*. J. Neurochem.

[199] Mogi M., Harada M., Kondo T., Riederer P., Inagaki H., Minami M., Nagatsu T. (1994). Interleukin-1 beta, interleukin-6, epidermal growth factor and transforming growth factor-alpha are elevated in the brain from parkinsonian patients. Neurosci. Lett.

[200] Sriram K., Matheson J.M., Benkovic S.A., Miller D.B., Luster M.I., O’Callaghan J.P. (2002). Mice deficient in TNF receptors are protected against dopaminergic neurotoxicity: Implications for Parkinson’s disease. FASEB J.

[201] Hayden M.S., Ghosh S. (2004). Signaling to NF-kappaB. Genes Dev.

[202] Saggu H., Cooksey J., Dexter D., Wells F.R., Lees A., Jenner P., Marsden C.D. (1989). A selective increase in particulate superoxide dismutase activity in parkinsonian substantia nigra. J. Neurochem.

[203] Tsay H.J., Wang P., Wang S.L., Ku H.H. (2000). Age-associated changes of superoxide dismutase and catalase activities in the rat brain. J. Biomed. Sci.

[204] Pearce R.K., Owen A., Daniel S., Jenner P., Marsden C.D. (1997). Alterations in the distribution of glutathione in the substantia nigra in Parkinson’s disease. J. Neural. Transm.

[205] Sofic E., Lange K.W., Jellinger K., Riederer P. (1992). Reduced and oxidized glutathione in the substantia nigra of patients with Parkinson’s disease. Neurosci. Lett.

[206] Huang C.S., Anderson M.E., Meister A. (1993). Amino acid sequence and function of the light subunit of rat kidney gamma-glutamylcysteine synthetase. J. Biol. Chem.

[207] Huang C.S., Chang L.S., Anderson M.E., Meister A. (1993). Catalytic and regulatory properties of the heavy subunit of rat kidney gamma-glutamylcysteine synthetase. J. Biol. Chem.

[208] Lu S.C., Huang Z.Z., Yang H., Tsukamoto H. (1999). Effect of thioacetamide on the hepatic expression of gamma-glutamylcysteine synthetase subunits in the Rat. Toxicol. Appl. Pharmacol.

[209] Zhu Y., Carvey P.M., Ling Z. (2006). Age-related changes in glutathione and glutathione-related enzymes in rat brain. Brain Res.

[210] Vermulst M., Bielas J.H., Kujoth G.C., Ladiges W.C., Rabinovitch P.S., Prolla T.A., Loeb L.A. (2007). Mitochondrial point mutations do not limit the natural lifespan of mice. Nat. Genet.

[211] Schriner S.E., Linford N.J., Martin G.M., Treuting P., Ogburn C.E., Emond M., Coskun P.E., Ladiges W., Wolf N., van Remmen H. (2005). Extension of murine life span by overexpression of catalase targeted to mitochondria. Science.

[212] Perier C., Bove J., Dehay B., Jackson-Lewis V., Rabinovitch P.S., Przedborski S., Vila M. (2010). Apoptosis-inducing factor deficiency sensitizes dopaminergic neurons to parkinsonian neurotoxins. Ann. Neurol.

